# Making Change in a Clinical Training Environment: A Checklist to Discuss the Process

**DOI:** 10.5334/pme.2410

**Published:** 2026-05-21

**Authors:** Fedde Scheele, Corry den Rooyen

**Affiliations:** 1 Faculty of Science, Vrije Universiteit Amsterdam, Amsterdam, The Netherlands; 2 Academic Centre for Dentistry Amsterdam (ACTA), a joint institute of the University of Amsterdam and Vrije Universiteit Amsterdam, Amsterdam, The Netherlands; 3 Training and Coaching Center for Clinical Educators and Program Directors (De Tweede Praktijk), Lent, The Netherlands

## Abstract

**Background & Need for Innovation:**

Effective change management is critical to the success of organizational change initiatives. Failure rates are estimated at approximately 70%. Change projects in clinical education are embedded in complex environments, where patient care has priority, professionals traditionally enjoy a high degree of autonomy, and multiple stakeholders are involved. Achieving successful change in such settings requires careful preparation by leaders. External consultancy support is costly, while gaining a comprehensive overview of the change management literature is time-consuming.

**Goal of Innovation:**

A practical tool that prompts relevant questions tailored to clinical training settings.

**Steps taken for Development and Implementation of Innovation:**

The checklist was developed using a developmental evaluation approach. Over several years, we applied a question-based tool grounded in key concepts from the change management literature. Initially consisting of twelve questions, the tool was iteratively expanded based on user experiences, identifying themes most relevant to clinical training contexts. Multiple-choice response options were developed to prevent unproductive discussions.

**Outcomes of Innovation:**

The final product is a twenty-item checklist designed for discussion in small groups. Ideally, different stakeholder groups complete the checklist separately and subsequently compare perspectives on the change process. This approach facilitates a shared understanding of the risks of the change initiative and highlights unaddressed tasks. A game board, cards, and instructions are available for download and printing.

**Critical Reflection on Our Process:**

This change management tool offers a structured journey through selected change management concepts, supporting leaders in clinical training environments and stimulating dialogue about change processes in clinical contexts.

## Background & Need for Innovation

In the literature on planned organizational change, failure rates are frequently reported to be as high as approximately 70% [1,2]. Although this estimate is difficult to establish with methodological rigor, the inherently challenging nature of change initiatives is well recognized and reflected in our own experience with the modernization of postgraduate training [3]. These failure rates appear to be particularly pronounced in settings characterized by strong professional autonomy, such as hospitals and other clinical environments [4]. Medical specialties are characterized by highly specific content, distinct clinical environments, and deeply entrenched hierarchical cultures [5], rendering meaningful change particularly challenging. From a complex adaptive systems perspective, change in such contexts may be facilitated through collaborative, multidisciplinary, and multiprofessional communities that engage in the shared negotiation of values and authority through open dialogue [4,5]. Change in clinical contexts is therefore not a mechanistic process, but one that is further complicated by internal political and cultural dynamics [4,5,6,7], in addition to societal pressures to, for example, deliver high-value, cost-conscious care.

Leading change in clinical environments is further complicated by a common professional misconception that high-quality ideas or products will “sell themselves” [8]. This belief may lead to an underestimation of the importance of a well-designed change process. Its effects may be exacerbated by the Dunning–Kruger effect [9], particularly when health professionals do engage with change management but overestimate their competence in this domain. In such cases, there is a heightened risk of oversimplifying the change process, potentially leading to failure. Another consequence of the Dunning–Kruger phenomenon is insufficient attention to reframing overly ambitious change initiatives. Projects must be appropriately scoped to remain achievable [4,10]. Large initiatives, such as “implementing a competency-based curriculum”, are often better divided into smaller, attainable components that can be sequenced to align with the existing curriculum and organizational context. Distal goals set direction; proximal goals regulate action [10].

To adopt a more robust approach, health professionals may require support from change management specialists or need to engage directly with the change management literature. However, the use of external expertise is often constrained by limited financial resources. Moreover, the change management literature is extensive and requires careful alignment with context, stakeholder configuration, and problem definition. Kuipers et al. [7] demonstrate that change approaches are highly context dependent, highlighting, for example, differences between private and public organizations in professional autonomy, political structures, legal constraints, and competing goals. Widely cited universalistic change models, such as those proposed by Kotter [1] and Lewin, often insufficiently account for clinical culture and institutional complexity, thereby overstating the general applicability of prescriptive, linear approaches [11].

Furthermore, designing a change plan in a multistakeholder context such as the clinic, particularly in cultures characterized by lower power distance [12], depends not only on insights from the literature but also on dialogue with the stakeholders involved. Empirical evidence shows that stakeholder participation in design processes improves change outcomes [13].

Health professionals engaged in change initiatives commonly encounter the following challenges:

1. Identifying all relevant stakeholders and actively involving them.
2. Defining, through dialogue, an initial change objective of manageable scope and, where appropriate, subsequent objectives.
3. Organizing sessions for each stakeholder group to address context-appropriate change management questions, resulting in a concrete task list for the project.
4. Comparing the outcomes of stakeholder sessions and building consensus through dialogue; the resulting task list should emerge from co-creation rather than unilateral decision-making [14].
5. Structuring the task list in terms of responsible actors, budgets, and timelines.
6. Initiating the change process, supported by regular progress updates, dialogue on improvement, and the integration of new insights within an ongoing learning cycle.

## Goal of Innovation

This article addresses the third challenge: the use of questions derived from change management literature that are tailored to the context. We describe the development of a structured list of questions based on both the literature and our experience with clinical change processes. The checklist is intended to provide users with insight into the complexity of their change initiatives while simultaneously offering a practical task list to guide implementation. This enhanced understanding highlights potential risks of failure and may, at times, indicate the need to reduce the scope of the proposed change. It also identifies key issues requiring attention, thereby generating concrete implementation tasks. Although the use of the checklist does not guarantee success, nor does it necessarily obviate the need for professional consultancy, it provides readily accessible guidance that may assist users in anticipating important pitfalls and considerations during the change process. The checklist is not intended to refine or replace existing change management frameworks; rather, it offers a concise set of key questions tailored to the clinical context.

## Steps taken for Development and Implementation of Innovation

### Setting

From 2003 onwards, the Dutch government initiated a national modernization of postgraduate medical specialty training. Substantial expertise in adult learning was already available within medical schools and general practice training programs, and insights from Maastricht University provided a strong foundation in medical education and research. During a period of strong governmental interest and investment, we were involved in at least ten nationwide change programs. These initiatives addressed topics such as innovative curriculum design for postgraduate training; learning from early implementations in obstetrics and gynaecology and paediatrics, and dissemination of curricular experiences across other medical specialties; legislative reform of postgraduate training; the introduction of market mechanisms for publicly funded training; training in high-value, cost-conscious care; preparation for an aging population; personalized training trajectories and the transition away from time-based training; and interprofessional education. In addition, we were involved in various “train-the-trainer” initiatives, including portfolio use, entrustment-based assessment, adult instruction, and the integration of the CanMEDS roles.

We participated in these processes at both national and local levels in our respective roles as gynaecologist and professor of health systems innovation and education (FS), and educational consultant (CdR). FS also served as project leader of a European modernization initiative within gynaecology.

### Developmental evaluation

The checklist was developed using a developmental evaluation approach, informed by principles of utilization-focused evaluation [15]. Designing state-of-the-art curricula or conceptual frameworks, for example, for training in high-value, cost-conscious care, proved complex but achievable. Translating these concepts into everyday clinical practice, however, constituted a considerably more challenging learning experience. Each project required a formal change plan as part of the funding application process. Throughout implementation, we continuously monitored successes and failures. Through dialogue, both among ourselves and within communities of learners [3], and informed by ongoing engagement with the literature, we sought to learn and adapt in an agile manner.

### Reflection, Reading, and Research

Each project was accompanied by continuous reflection and scientific research, usually within a PhD trajectory, during which questions related to change management frequently emerged. These questions were initially explored through careful engagement with the change management literature, seeking guidance applicable to the context. Literature explicitly addressing change management in healthcare training settings was scarce [16,17]. Authors who strongly influenced our thinking included Rogers [9], De Caluwé [18], Weggeman [19], Kotter [1], and Morgan [6]. Over time, through extensive reading and experiential learning, we gradually developed greater conceptual oversight while remaining aware of the limitations of our perspective. Notably, many colleagues readily engaged in dialogue about curricular content and assessment but were less inclined to engage similarly in the change process itself.

### Workshops with Clinicians and Nurses

In addition to content-focused workshops on portfolios, assessment, instruction, and feedback, we began offering workshops on change management. These sessions aimed to facilitate perspective change through small-group work on participants’ own cases, supported by key concepts from the literature. Across dozens of workshops, primarily in the Netherlands but also in other European countries and Canada, we identified which concepts resonated most strongly with clinicians. We believe that the pedagogical choices we made were well aligned with the European and Canadian clinical context. Our overarching aim was to support participants in becoming self-reliant change agents by equipping them with practical tools to apply change management knowledge within their own professional environments and stakeholder networks.

### Construction of the Checklist

Inspired by a game-based approach to accreditation [20], which increased the accessibility of a traditionally less engaging topic through the use of multiple-choice questions, we developed a similar instrument. The checklist initially comprised twelve questions and was expanded over the past decade to a total of twenty items. These questions are intended to elucidate the complexity of a given context or the specific areas in which action is required. The use of predefined response options serves to limit unproductive or excessively prolonged discussions.

The checklist has been tested with several hundred workshop participants, including more than 350 medical specialists, over 70 specialist trainees, and over 100 professionals in quality and administrative roles, and has been refined through an iterative process. In nearly all applications, the tool stimulated active and lively discussions, with full completion typically requiring up to 90 minutes. It is available online in an earlier Dutch version via the Dutch Federation of Medical Specialists, and we have used feedback from users. For example, in a later version, the sequence of questions was reorganized to reflect a more coherent timeline. Furthermore, the checklist has undergone narrative evaluation for content and face validity by at least ten experts. We considered it timely to disseminate this tool to an international audience, both to support practical implementation and to foster scholarly discussion on approaches to improving the quality of change management in clinical training settings.

### Game-based approach

The checklist functions as a structured, facilitated small-group intervention aimed at assessing and aligning stakeholders’ judgments of challenges and preparedness for change. Stakeholder groups complete an identical set of forced-choice items, negotiate consensus for each response, and subsequently compare responses across groups. This design activates several mechanisms relevant to implementation processes: (1) elicitation of tacit knowledge, by requiring participants to articulate implicit assumptions about current practices and preparedness for change; (2) structured deliberation, whereby consensus-building promotes reflection, perspective-taking, and collective sense-making; and (3) feedback on alignment, as comparison across stakeholder groups makes variation in interpretations and priorities visible. The use of constrained response options and consensus requirements functions as a facilitation strategy that lowers cognitive and social barriers to participation while structuring interaction. These “productive constraints” help transform individual perceptions into shared, discussable representations, thereby supporting the co-creation of an actionable understanding of implementation challenges. By surfacing discrepancies across stakeholder groups, the activity enables teams to identify risks and gaps in preparedness and to specify targeted preparatory actions (e.g., reframing the scope of the change initiative, addressing resource constraints, or aligning strategies), thereby supporting more effective implementation planning.

A game board, playing cards, a printable version of the checklist with options, and instructions for use are provided in the supplement.

## Outcomes of Innovation

The outcome is the change checklist, which is presented and explained in Table 1. In Box 1 two examples of user experiences are described.

**Table 1:** The change management checklist comprises 20 questions with corresponding response options intended to facilitate discussion. For each question, the checklist highlights both the underlying challenge and potential actions when the issue falls within the respondent’s sphere of influence. A brief rationale explaining the relevance of each topic is provided below each question.


Each question concerns risk assessment associated with specific choices and the extent to which one has influence over this aspect of the change. Items that address topics often within your control are marked with an icon to highlight areas where action may be required.	

**Question 1** What is the reason behind the change?	**Options:** a. It is a broadly shared ambition b. It is our personal ambition, but our coalition of colleagues with the same ambition is small c. It is necessary within our internal improvement system d. Our leadership commissioned it e. It is driven by external regulation

**Rationale** When the rationale for change is grounded in a broad stakeholder support, the change tends to be stronger. Within the Dutch cultural context, change management may become more challenging across responses A through E [1,19].	

**Question 2** Who commissioned the change?	**Options:** a. Our department b. A hospital committee c. The hospital board d. A national official body e. The central government

**Rationale** The extent to which a change initiative originates from one’s own deliberate choice is positively associated with the likelihood of its successful implementation. In this context, change management may again become more challenging across responses A through E [18,19].	

**Question 3** How complex is the change?	**Options:** a. Simple: The change does not adversely affect other systems or people. b. Complicated: It affects several systems and may disadvantage some people. c. Complex: The change affects multiple systems and groups of people with uncertain effects. d. Disruptive: It may induce chaos while attempting to solve a complex problem.

**Rationale** As the complexity of a change initiative increases, a greater number of stakeholders tend to become involved, thereby intensifying the challenges of implementation and reducing the likelihood of successful outcomes [4,8,10]. Could the level of complexity be reduced by reframing the overarching change objective into smaller sub-objectives?	

**Question 4** Do we trust that we can make this change work?	**Options:** a. Yes b. We will probably succeed c. Limited trust d. No trust

**Rationale** Rogers was a pioneer in the study of change as a phenomenon with distinct features. Higher levels of trust are associated with a greater likelihood of success in change initiatives [8].	

**Question 5** How much time will it take before this change is embedded in our way of working?	**Options:** a. <1 year b. 1–3 years c. 3–5 years d. >5 years

**Rationale** Organizational change requires substantial effort, particularly with regard to the development and adoption of new routines, and typically unfolds over an extended period. As the number of routines requiring modification increases, the time needed for the objectives of the change initiative to become fully institutionalized likewise increases. In such cases, because the risk of unsuccessful implementation may increase, it is advisable to reframe the overarching change objective into smaller, more manageable sub-objectives [8,10].	

**Question 6** Rogers distinguishes several adopter categories within a change process, ranging from innovators and early adopters to the early majority, late majority, and, finally laggards, who are the last to adopt an innovation. Within the context of your change initiative, which of these adopter groups are already represented?	**Options:** a. Innovators b. a + Adaptive people c. a + b + Early majority d. a + b + c + Late majority e. a + b + c + d + Laggards

**Rationale** Based on diffusion of innovation theory, the successful implementation of change relies on achieving a critical mass of adopters. At the individual level, this means that stakeholders must be willing to accept and put the innovation into practice. At the collective level, this threshold is generally reached when adoption spreads beyond innovators and early adopters to encompass the early majority. Once the early majority becomes involved, the diffusion process typically gains momentum, with later adopter groups following gradually. In contrast, if adoption remains limited to innovators, substantial additional effort and resources are needed to stimulate wider adoption and advance the change toward this critical mass. In clinical settings, professional groups often differ in their level of influence, which in turn affects the success of change initiatives. For example, even if all nurses support a change, a lack of support from the much smaller group of physicians can still hinder progress. Therefore, success depends not only on the number of adopters but also on their influence [8].	

**Question 7** Are both leadership and management positioned to support the change?	**Options:** a. Leadership: Yes or No b. Management: Yes or No

**Rationale** From the perspective of leadership and change theory, the successful implementation of a change initiative depends on the complementary roles of leadership and management. Leadership plays a critical role in articulating a compelling vision and setting strategic direction, often described as doing the right things, whereas management is responsible for translating this vision into coordinated action by ensuring efficiency and effectiveness in execution, or doing things right [1].	

**Question 8** To what extent does the team responsible for leading the change demonstrate sufficient diversity of competencies across domains such as political acumen, strategic planning, people management, adaptive learning, philosophical reflection, and crisis management?	**Options:** a. Political acumen: Yes or No b. Planning skills: Yes or No c. People management skills: Yes or No d. Learning skills to support trial and error: Yes or No e. Philosophical skills to be self-critical: Yes or No f. Skills for crisis management: Yes or No

**Rationale** An effective team for a change project requires a complementary mix of competencies distributed across its members. Core competencies typically include political acumen, strategic planning and execution, and people management. In addition, the capacity for adaptive learning through trial and error, as well as reflective engagement with the broader purpose and implications of the project from a more philosophical perspective, provides important added value. Finally, the ability to adopt an appropriate management approach during periods of crisis is, in some cases, essential for sustaining the change process [18].	

**Question 9** Are stakeholders affected by the change adequately motivated to support its implementation?	**Options:** a. Yes b. Partially c. No

**Rationale** Employee motivation is commonly viewed as a central component of readiness for change and has been consistently linked to successful outcomes in organizational change efforts [16]. Within hospital settings, stakeholders can be categorized into various groups, such as clinical specialties (e.g., internal medicine, surgery, radiology), professional disciplines (e.g., physicians, nurses, managers), and roles such as administrative and support staff. Although these groups are often interdependent, change initiatives frequently overlook stakeholders whose influence is perceived as less significant.	

**Question 10** Do the stakeholders affected by the change possess the required training and competencies?	**Options:** a. Yes b. Partially c. No

**Rationale** Employee change readiness constitutes a critical enabling condition for the successful implementation of organizational change. While employees’ understanding of and motivation for the change are important components of readiness, the capacity to effectively perform the tasks required by the change is equally essential. Accordingly, sustained attention to training and professional development is necessary to support effective change execution [1,16].	

**Question 11** Are material resources like time, money and equipment well arranged?	**Options:** a. Yes b. Partially c. No

**Rationale** Kotter’s change framework underscores the importance of removing barriers to action, including ensuring that sufficient time, financial resources, and material support are available to facilitate successful change outcomes. From a diffusion-of-innovation perspective, the availability of adequate time, financial means, and material infrastructure supports trialability and reduces perceived complexity, both of which are critical determinants of successful innovation adoption [1].	

**Question 12** Which statement best describes professionals affected by the change?	**Options:** a. Professionals are already involved in its design. b. Professionals are involved in evaluation and improvement. c. Professionals only execute the change.

**Rationale** Change management in clinical settings must explicitly recognize the professional context in which it is embedded. To foster support and enhance the likelihood of successful outcomes, it is essential to involve professionals at an early stage by providing them with meaningful opportunities to influence the design, implementation, and iterative adaptation of initiatives based on monitoring and evaluation findings [4,7,14,16,19].	

**Question 13** Do we sufficiently care for all stakeholders affected by the change?	**Options:** a. Yes b. Partially c. No

**Rationale** Careful consideration of the side effects experienced by groups affected by change is essential for fostering support and enhancing the likelihood of successful change outcomes [18].	

**Question 14** How detailed are the instructions for implementing the change?	**Options:** a. Very loosely defined, since change is expected to happen mainly organically. b. A broad overview with room for professionals to fill in. c. Very detailed, all affected parties know exactly what to do.

**Rationale** Professionals generally value autonomy and trialability, particularly the opportunity to identify and implement solutions in ways they consider most appropriate for achieving agreed-upon objectives. Excessively detailed instructions, although sometimes requested, may have a demotivating effect and undermine professional engagement. Conversely, reliance on entirely organic change processes entails significant risk, as it raises the question of why such change would emerge at this moment if it has not occurred previously. Accordingly, we recommend a careful analysis of the organizational context to determine an appropriate level of instructional detail that balances guidance with professional autonomy [4,18,19].	

**Question 15** How do affected professionals generally judge profit versus loss due to the change?	**Options:** a. Profit > loss b. Profit and loss in equilibrium c. Loss > profit

**Rationale** Kahneman showed that individuals tend to be highly sensitive to experiences of loss, whereby even relatively minor perceived losses can outweigh larger anticipated gains. Consequently, when the perceived losses associated with a change initiative exceed the perceived or expected benefits, the probability of successful implementation decreases. In particular, losses perceived by influential stakeholders like physicians may pose a significant challenge in a change project that requires careful people management [18].	

**Question 16** To what extent does the organization foster an open culture of feedback and improvement, and possess the capacity to address critical voices in a constructive manner?	**Options:** a. Yes b. Partially c. No

**Rationale** Agile change approaches emphasize the pursuit of a clearly articulated goal in combination with iterative cycles of feedback, critical reflection, and adaptation. Such continuous learning processes allow organizations to respond effectively to uncertainty and complexity, thereby enhancing the effectiveness of change initiatives [4,14,18].	

**Question 17** During the change, speaking up is rewarded.	**Options:** a. Yes b. Partially c. No

**Rationale** Amy Edmondson conceptualized psychological safety as a shared belief that individuals can speak up, share ideas, and take interpersonal risks without fear of negative consequences. Empirical evidence from Google (Project Aristotle) demonstrated that psychological safety was a key enabling factor for team effectiveness and innovative performance. On this basis, we recommend that organizations actively encourage and reward employee voice, including the expression of ideas, perspectives, and strategic visions. The establishment of a psychologically safe environment constitutes a necessary precondition for fostering such a culture [4,13,14,21].	

**Question 18** How is resistance to change addressed?	**Options:** a. Through dialogue and room for individual choices b. Through dialogue and persuasion c. Through enforcement of the mandatory change

**Rationale** Kotter’s framework addresses resistance to change through the creation of urgency, the establishment of a guiding coalition, the communication of a clear vision, and the removal of structural and psychological barriers. Nevertheless, resistance may persist, necessitating the deliberate selection of an appropriate communication strategy. Dialogical approaches are generally preferable, particularly in professional contexts characterized by high levels of autonomy, such as hospital settings. Dialogue supports vision alignment and promotes behavioral change grounded in shared understanding and commitment. In contrast, enforcement may result in compliance without internalized acceptance, increasing the risk of superficial adherence and circumvention of the intended change. Consequently, enforcement is typically regarded as a strategy of last resort. While it may be justified in situations of high urgency, legal obligation, or persistent obstruction rooted in vested interests, its use entails risks to trust, commitment, and psychological safety and should therefore be applied selectively, proportionately, and in combination with clear communication and supportive measures [1,13,14,18,19].	

**Question 19** What monitoring indicators are used?	**Options:** a. Contented customers b. Professionals executing the change-related related routines c. Professionals able to execute the change d. Contented Professionals

**Rationale** Within the Plan-Do-Check-Act cycle, systematic monitoring constitutes a central mechanism for organizational learning and adaptive improvement. During the Check phase, performance is assessed against intended objectives; however, in practice, monitoring frequently relies on proxy indicators rather than direct measures of the ultimate change goals. Indicators that more closely approximate the intended outcomes enhance the validity of evaluation and support more informed decision-making in the Act phase. Nevertheless, when direct measurement is not feasible, the pragmatic use of well-chosen proxy measures represents an acceptable and often necessary approach to sustaining iterative learning and continuous improvement.	

**Question 20** How is the change consolidated?	**Options:** a. Trust in professional ownership b. Through established routines on the work floor c. By rewarding the desired behavior d. Through management-driven monitoring and improvement cycles e. Through external monitoring supported by legislation

**Rationale** In healthcare settings, the sustainability of change depends on the extent to which new practices become normalized within everyday clinical work. Drawing on Normalization Process Theory, consolidation is understood as a social and collective process through which clinicians integrate new ways of working into existing professional routines. This process requires shared sense-making regarding the purpose and value of the change (coherence), sustained professional engagement and ownership (cognitive participation), alignment of roles, workflows, and resources (collective action), and ongoing appraisal of the change in practice (reflexive monitoring). In highly professionalized healthcare environments, normalization is facilitated by trust, professional autonomy, and the compatibility between the change and established clinical routines. Where these conditions are insufficiently addressed, change initiatives are likely to remain fragile and vulnerable to erosion or reversal over time.

**Box 1 Two examples of user experiences**

Vignette 1 relates to question 8

Implementing Entrustable Professional Activities

Medical educators within a specialty training program planned to introduce newly described Entrustable Professional Activities (EPAs) into their local curriculum. Initial discussions focused on leadership considerations, particularly how EPAs might strengthen the training program and benefit residents and clinical educators. The team expressed strong commitment to the initiative.

When reviewing the implementation checklist, the educators recognized that several complementary roles had received limited attention. In particular, the role of the *planner* had been overlooked. The team acknowledged the need for a structured implementation plan, including clear goals, timelines, and allocation of responsibilities. They also recognized the importance of the *people manager* role in ensuring that residents would perceive the changes as meaningful in daily training.

In retrospect, guided by the checklist’s emphasis on adaptive learning, the educators could have decided to begin with a pilot and approach implementation as an iterative learning cycle. They also could have invited a critical colleague in the role of philosopher, to challenge underlying assumptions about the expected benefits.

This reflection highlighted that approaching implementation primarily from a leadership perspective may overlook other roles essential for successful educational change.

Vignette 2 relates to question 9

Introducing Interprofessional Handover

A medical team aimed to strengthen interprofessional collaboration within residency training. As an initial step, the team proposed combining the medical and nursing handovers and adjusted the timing of the medical handover to enable joint participation. The change was intended to create opportunities for residents to engage in interprofessional practice during routine clinical work.

While reviewing the implementation checklist, the team reflected on question 9 and questioned whether nurses had been sufficiently positioned as key stakeholders. Although the initiative aimed to promote interprofessional collaboration, the planning had largely been undertaken by the medical team.

Subsequent discussions with nursing staff revealed that further dialogue was needed about the meaning and goals of interprofessional collaboration. Physicians and nurses appeared to attribute different values and expectations to the concept.

This reflection underscored the importance of early stakeholder engagement and developing understanding when implementing interprofessional educational initiatives.

### Critical reflection on the process and outcome

The change management checklist guides users through selected change management literature by linking the specific change project under consideration to twenty relevant process-oriented questions. Through a game-based approach that stimulates lively group discussions and requires consensus for each question, professionals are encouraged to engage in deliberations that often yield unexpected perspectives. As noted earlier, professionals tend to focus primarily on content-related discussions, such as defining a set of Entrustable Professional Activities (EPAs), while comparatively little attention is paid to the processes involved in implementing such a set. Given the vulnerability of implementation phases, the concise nature of the checklist and the facilitated dialogue on change processes may substantially contribute to the successful realization of innovations.

One of the challenges encountered during the development of the checklist was the incorporation of cultural aspects. In certain change processes, even when most prerequisites appear to be met, intangible forces may still impede progress. Culture can exert influence at multiple levels. At the macro level, for example, differences between countries such as the Netherlands and Indonesia may reflect contrasting orientations toward individualism and collectivism [12]. At the meso level, cultural influences may emerge through differing valuations of intensive involvement in clinical training versus high-level research, particularly when comparing general and academic hospitals. Cultural differences between physicians and nursing staff may also be impactful. At the micro level, within everyday work environments, subtle variations in psychological safety [21] or longstanding unresolved issues may have unforeseen effects.

The checklist may identify accumulating risk factors in a change initiative. When the overall risk of failure becomes substantial, particularly if key factors lie outside the influence of those involved, the checklist alone cannot ensure success. In such cases, the initiative may need to be reconsidered or reframed into a more achievable form. While such reframing can restore more favourable prospects for change, facilitating it lies beyond the scope of the checklist.

The checklist is not intended as a prescriptive guide for managing change projects, but rather as a tool to facilitate dialogue through the deliberate selection of topics derived from change management theory. Nevertheless, users should remain attentive to intangible cultural factors and to indications from the risk assessment that reframing the initiative may be necessary.

In clinical environments, power distance between physicians, nurses, and other allied health professionals may be substantial, which can impede productive dialogue in small groups composed of mixed professions. In such cases, we recommend organizing groups by profession and subsequently comparing the results in a facilitated discussion led by an independent moderator.

Furthermore, as the checklist was developed within a Western context—where hierarchical dynamics may influence interaction—its validity may be reduced in markedly different cultural settings. In such contexts, other cultural dimensions may complicate dialogue [12], and additional or alternative questions may need to be considered.

The checklist has undergone content and face validation by several experts. However, its effectiveness in supporting change projects in clinical contexts warrants further investigation. Additional research questions include its robustness across markedly different cultural contexts, such as those in Asia or Africa, and potential comparisons with other change management checklists for clinical training, which we have unfortunately not yet been able to identify. At present, one of the most salient questions for the authors concerns whether the implementation of the checklist within organizations will lead to more frequent and structured change management dialogues.

## Conclusion

The change management checklist is a support tool for change initiatives, primarily situated within the clinical context. It has been developed through iterative application and systematic refinement, informed by both the literature and extensive user experience. Its use has proven engaging and has yielded meaningful insights, suggesting potential value for readers. In addition, we aim to stimulate further discussion within the medical education literature, as sustained scholarly attention to change management in clinical training remains essential given the inherent vulnerability of implementation processes.

## Statement on AI use

ChatGPT (version 5.2) was used for language editing to improve grammar and readability. No content was generated by AI; all intellectual content and interpretations are those of the authors. The use of AI was limited to supporting clarity and accessibility for an international audience.

## Additional Files

The additional files for this article can be found as follows:

10.5334/pme.2410.s1 Supplement 1a. Printable game board for the change.

10.5334/pme.2410.s2 Supplement 1b. Printable cards for the game-digital.

10.5334/pme.2410.s3 Supplement 1c. How to Use the Checklist Game Board.

10.5334/pme.2410.s4 Supplement 1d. Table 1 and printable version.
