# Supplementary material for: Making Change in a Clinical Training Environment: A Checklist to Discuss the Process
Source: Perspect Med Educ. 2026 May 21;15(1):449–59. doi: 10.5334/pme.2410 (PMC13196687; doi:10.5334/pme.2410)
Supplement: Supplement 1a. — Printable game board for the change. [file pme-15-1-2410-s1.pdf]

1

What is the reason
behind the change?

Choose 1 option and place the card
of your choice on the game board

1

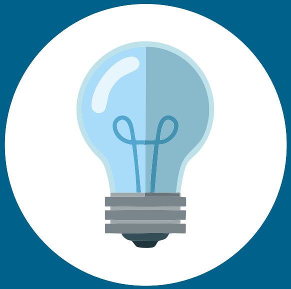

2

Who commissioned
the change?

Choose 1 option and place the card
of your choice on the game board

2

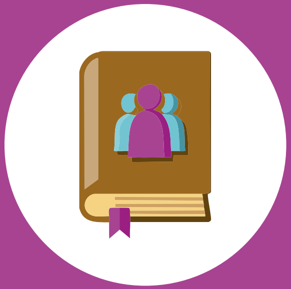

3

How complex
is the change?

Choose 1 option and place the card
of your choice on the game board

3

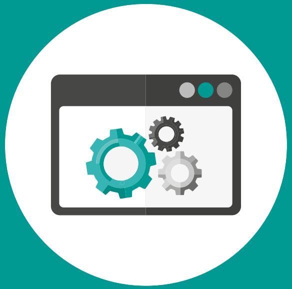

4

Do we trust that we can
make this change work?

Choose 1 option and place the card
of your choice on the game board

4

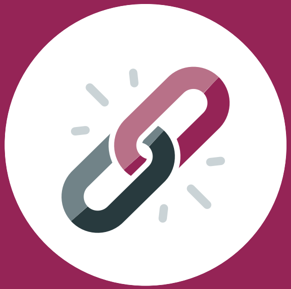

9

Are stakeholders affected by the
change adequately motivated
to support its implementation?

Choose 1 option and place the card
of your choice on the game board

9

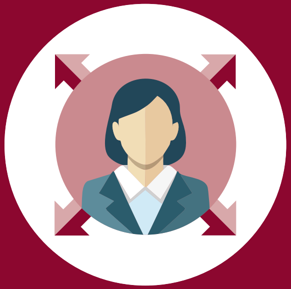

T H

10

Do the stakeholders affected by
the change possess the required
training and competencies?

Choose 1 option and place the card
of your choice on the game board

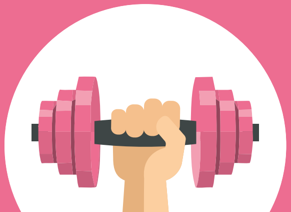

5

How much time will it take before this change is embedded in our way of working?

Choose 1 option and place the card of your choice on the game board

5

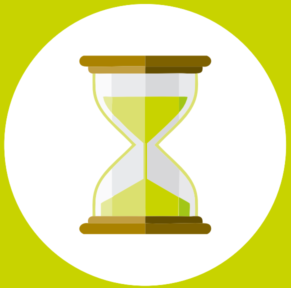

6

Rogers distinguishes several adopter categories within a change process, ranging from innovators and early adopters to the early majority, late majority, and, finally laggards, who are the last to adopt an innovation. Within the context of your change initiative, which of these adopter groups are already represented?
(You may choose more than one option)

Place up to 5 cards on the game board

6

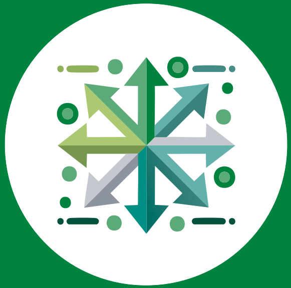

7

Are both leadership and management positioned to support the change?

Place up to 2 cards on the game board

7

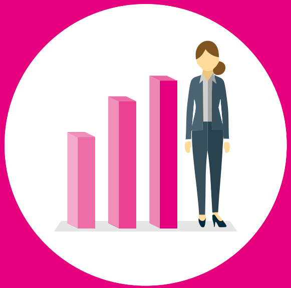

8

To what extent does the team responsible for leading the change demonstrate sufficient diversity of competencies across domains such as political acumen, strategic planning, people management, adaptive learning, philosophical reflection, and crisis management?

Place up to 5 cards on the game board

8

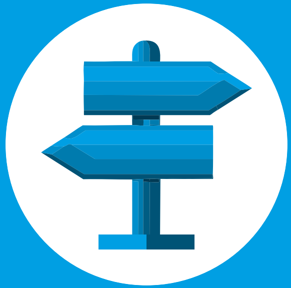

E GAME

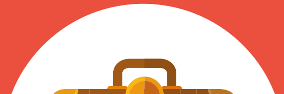

11

10

C H A N G

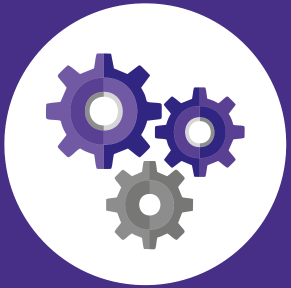

13

Do we sufficiently care for all stakeholders affected by the change?

Choose 1 option and place the card of your choice on the game board

13

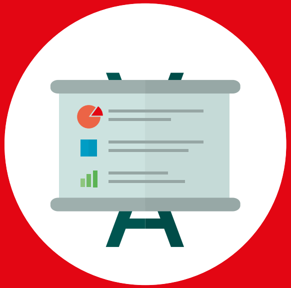

14

How detailed are the instructions for implementing the change?

Choose 1 option and place the card of your choice on the game board

14

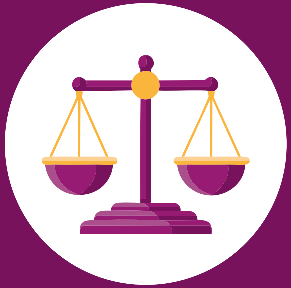

15

How do professionals generally evaluate the balance between gains and losses due to the change?

Choose 1 option and place the card of your choice on the game board

15

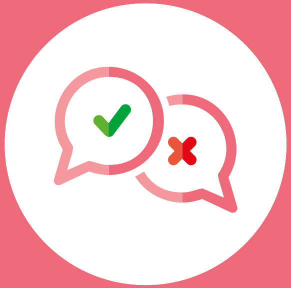

16

To what extent does the organization foster an open culture of feedback and improvement, and possess the capacity to address critical voices in a constructive manner?

Choose 1 option and place the card of your choice on the game board

16

G E R

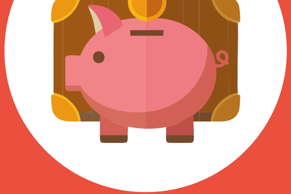

Are material resources like time, money and equipment well arranged?

Choose 1 option and place the card of your choice on the game board

11

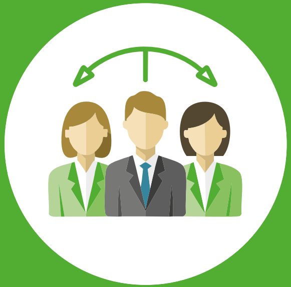

12

Which statement best describes professionals affected by the change?

Choose 1 option and place the card of your choice on the game board

12

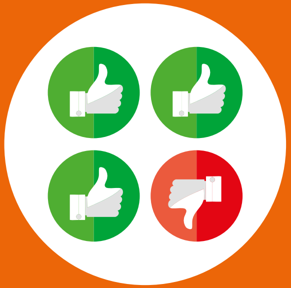

17

During the change, speaking up is rewarded.

Choose 1 option and place the card of your choice on the game board

17

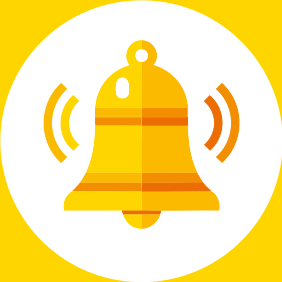

18

How is resistance to change addressed?

Choose 1 option and place the card of your choice on the game board

18

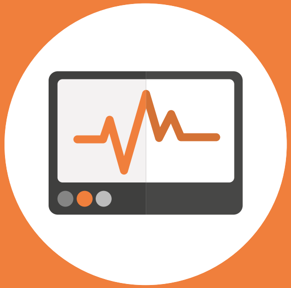

19

What monitoring indicators are used?

Choose up to 4 options and place cards on the game board

19

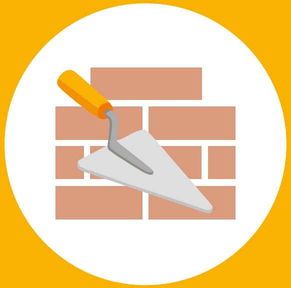

20

How is the change consolidated?

Choose up to 5 options and place card on the game board

20

© Copyright 2026 Corry den Rooijen - De Tweede Praktijk, prof.dr. Fedde Scheele.
