# Supplementary material for: Making Change in a Clinical Training Environment: A Checklist to Discuss the Process
Source: Perspect Med Educ. 2026 May 21;15(1):449–59. doi: 10.5334/pme.2410 (PMC13196687; doi:10.5334/pme.2410)
Supplement: Supplement 1b. — Printable cards for the game-digital. [file pme-15-1-2410-s2.pdf]

1

It is driven by external regulation

1

1

It is necessary within our
internal improvement system

1

1

Our leadership commissioned it

1

1

It is our personal ambition, but our coalition of colleagues with the same ambition is small

1

1

It is a broadly shared ambition

1

2

Our department

2

2

A hospital committee

2

2

The hospital board

2

2

A national official body

2

2

The central government

2

3

Simple
The change does not adversely affect other systems or people

3

3

Complicated
It affects several systems and may disadvantage some people

3

3

Complex
The change affects multiple systems and groups of people with uncertain effects

3

3

Disruptive
It may induce chaos while attempting to solve a complex problem

3

4

Yes

4

4

We will probably succeed

4

4

Limited trust

4

4

No trust

4

5

<1 year

5

5

1-3 years

5

5

3-5 years

5

5

>5 years

5

6

Innovators

6

6

a + Adaptive people

6

6

a + b + Early majority

6

6

a+ b + c + Late majority

6

6

a + b + c + d + Laggards

6

7

Leadership
Yes or No

7

7

Management
Yes or No

7

8

Political acumen
Yes or No

8

8

Planning skills
Yes or No

8

8

People management skills
Yes or No

8

8

Learning skills to support trial and error
Yes or No

8

8

Philosophical skills to be self-critical
Yes or No

8

8

Skills for crisis management
Yes or No

8

9

Yes

9

9

Partially

9

9

No

9

10

Yes

10

10

Partially

10

10

No

10

11

Yes

11

11

Partially

11

11

No

11

12

Professionals are already involved in its design

12

12

Professionals are involved in evaluation and improvement

12

12

Professionals only execute the change

12

13

Yes

13

13

Partially

13

13

No

13

14

Very loosely defined, since change is
expected to happen mainly organically

14

14

Just the bigger picture with
room for professionals to fill in

14

14

Very detailed, all affected parties
know exactly what to do

14

15

Profit > loss

15

15

Profit and loss in equilibrium

15

15

Loss > profit

15

16

Yes

16

16

Partially

16

16

No

16

17

Yes

17

17

Partially

17

17

No

17

18

Through dialogue and room
for individual choice

18

18

Through dialogue and persuasion

18

18

Through enforcement of
the mandatory change

18

19

Contented professionals

19

19

Professionals executing the
desired change-related routines

19

19

Professionals able to execute the change

19

19

Contented professionals

19

20

Trust in professional ownership

20

20

By inescapable routines on the work floor

20

20

By rewarding the planned behavior

20

20

By management driven monitoring
and improvement cycles

20

20

By external monitors supported by legislation

20

Disagreement card

Disagreement card

Disagreement card

Disagreement card

Disagreement card

THE GAME CHANGER

- Think of a planned change or visualize a change you would like to make.
- Select the number of options from the options given. Discuss the choosen options in your group.
- By doing so, you will get an idea of how complex a change process can be, which strategy you´d prefer and consider what's most effective in your situation.

© Copyright 2026 Corry den Rooijen -
De Tweede Praktijk, prof.dr. Fedde Scheele.

DE TWEEDE PRAKTIJK
