# Supplementary material for: Making Change in a Clinical Training Environment: A Checklist to Discuss the Process
Source: Perspect Med Educ. 2026 May 21;15(1):449–59. doi: 10.5334/pme.2410 (PMC13196687; doi:10.5334/pme.2410)
Supplement: Supplement 1c. — How to Use the Checklist Game Board. [file pme-15-1-2410-s3.pdf]

How to Use the Checklist Game Board

The checklist game board is designed as a facilitated group dialogue to support structured reflection on change initiatives.

For the first use of the game board: print the four parts of the board on A4 paper and tape them together in the order of the questions.

1. Organize the session.
   If possible, involve a facilitator and have multiple small groups (3–6 participants) work with the game board.
2. Prepare the materials.
   Place the game board on a table and position participants around it so that everyone can clearly see and read its content. Place the option cards next to the board. Each checklist question on the board is associated with several option cards. Provide participants with a printed checklist including explanations, so they have a clear overview of the available options.
3. Define the change initiative.
   As a group, select the change initiative you wish to discuss and formulate it as specifically as possible.
4. Start with the first question.
   Read question 1 on the game board and review the corresponding option cards.
5. Discuss the options.
   Discuss the available options within the group and explore how each relates to the proposed change initiative.
6. Select the most appropriate option(s).
   Choose the option(s) that best fit the change initiative and place them on the board. Although consensus is desirable, disagreement can be informative. When disagreement occurs, mark it with a disagreement card so that it can be revisited later when comparing outcomes with other groups. The arguments raised during discussion may provide insight into participants' assumptions and perspectives.
7. Continue through the checklist.
   Repeat steps 4–6 for all 20 questions. Working through the checklist helps participants develop a shared understanding of the complexity and potential risks of the change process, the strategies that may be most appropriate, and the tasks that emerge from reviewing the initiative.
8. Complete the checklist discussion.
   Aim to complete the discussion of the 20 questions within approximately 90 minutes.
9. Compare perspectives across groups.
   After completing the checklist, compare the outcomes across groups. Identify areas of agreement and divergence among stakeholders and explore the underlying reasons. This comparison may highlight topics requiring further dialogue and help identify additional actions needed for implementation.
10. Conclude the session.
    Allow approximately 30 minutes for the comparison phase. In most cases, the gamified dialogue can be completed within two hours, providing participants with an overview of key insights and potential next steps.

© 2026, Drs. Corry den Rooijen, De tweede praktijk, prof. dr. Fedde Scheele
