# Supplementary material for: Making Change in a Clinical Training Environment: A Checklist to Discuss the Process
Source: Perspect Med Educ. 2026 May 21;15(1):449–59. doi: 10.5334/pme.2410 (PMC13196687; doi:10.5334/pme.2410)
Supplement: Supplement 1d. — Table 1 and printable version. [file pme-15-1-2410-s4.pdf]

Checklist

Legend: The change management checklist comprises 20 questions with corresponding response options intended to facilitate discussion. For each question, the checklist highlights both the underlying challenge and potential actions when the issue falls within the respondent's sphere of influence. A brief rationale explaining the relevance of each topic is provided below each question.

Each question concerns risk assessment associated with specific choices and the extent to which one has influence over this aspect of the change. Items that adress topics often within your control are marked with an icon to highlight areas where action may be required.

**Question 1**<br>What is the reason behind the change?	**Options:**<br>a. It is a broadly shared ambition<br>b. It is our personal ambition, but our coalition of colleagues with the same ambition is small<br>c. It is necessary within our internal improvement system<br>d. Our leadership commissioned it<br>e. It is driven by external regulation
**Rationale:** When the rationale for change is grounded in a broad stakeholder support, the change tends to be stronger. Within the Dutch cultural context, change management may become more challenging across responses A through E.	
**Question 2**<br>Who commissioned the change?	**Options:**<br>a. Our department<br>b. A hospital committee<br>c. The hospital board<br>d. A national official body<br>e. The central government
**Rationale:** The extent to which a change initiative originates from one's own deliberate choice is positively associated with the likelihood of its successful implementation. In this context, change management may again become more challenging across responses A through E	
**Question 3**<br>How complex is the change? 	**Options:**<br>a. Simple: The change does not adversely affect other systems or people.<br>b. Complicated: It affects several systems and may disadvantage some people.<br>c. Complex: The change affects multiple systems and groups of people with uncertain effects.<br>d. Disruptive: It may induce chaos while attempting to solve a complex problem.
**Rationale:** As the complexity of a change initiative increases, a greater number of stakeholders tend to become involved, thereby intensifying the challenges of implementation and reducing the likelihood of successful outcomes. Could the level of complexity be reduced by reframing the overarching change objective into smaller sub-objectives?	
**Question 4**<br>Do we trust that we can make this change work?	**Options:**<br>a. Yes<br>b. We wil probably succeed.<br>c. Limited trust<br>d. No trust
**Rationale:** Rogers was a pioneer in the study of change as a phenomenon with distinct features. Higher levels of trust are associated with a greater likelihood of success in change initiatives.	
**Question 5**<br>How much time will it take before this change is embedded in our way of working? 	**Options:**<br>a. <1 year<br>b. 1-3 years<br>c. 3-5 years<br>d. >5 years
**Rationale:** Organizational change requires substantial effort, particularly with regard to the development and adoption of new routines, and typically unfolds over an extended period. As the number of routines requiring modification increases, the time needed for the objectives of the change initiative to become fully institutionalized likewise increases. In such	

<table>
<tr><td colspan="2">cases, because the risk of unsuccessful implementation may increase, it is advisable to reframe the overarching change objective into smaller, more manageable sub-objectives</td></tr>
<tr><td>Question 6
Rogers distinguishes several adopter categories within a change process, ranging from innovators and early adopters to the early majority, late majority, and, finally laggards, who are the last to adopt an innovation. Within the context of your change initiative, which of these adopter groups are already represented?</td><td>Options:
a. Innovators
b. a + Adaptive people
c. a + b + Early majority
d. a+ b + c + Late majority
e. a + b + c + d + Laggards</td></tr>
<tr><td colspan="2">Rationale: Based on diffusion of innovation theory, the successful implementation of change relies on achieving a critical mass of adopters. At the individual level, this means that stakeholders must be willing to accept and put the innovation into practice. At the collective level, this threshold is generally reached when adoption spreads beyond innovators and early adopters to encompass the early majority. Once the early majority becomes involved, the diffusion process typically gains momentum, with later adopter groups following gradually. In contrast, if adoption remains limited to innovators, substantial additional effort and resources are needed to stimulate wider adoption and advance the change toward this critical mass. In clinical settings, professional groups often differ in their level of influence, which in turn affects the success of change initiatives. For example, even if all nurses support a change, a lack of support from the much smaller group of physicians can still hinder progress. Therefore, success depends not only on the number of adopters but also on their influence.</td></tr>
<tr><td>Question 7
Are both leadership and management positioned to support the change?</td><td>Options:
a. Leadership: Yes or No
b. Management: Yes or No</td></tr>
<tr><td colspan="2">Rationale: From the perspective of leadership and change theory, the successful implementation of a change initiative depends on the complementary roles of leadership and management. Leadership plays a critical role in articulating a compelling vision and setting strategic direction, often described as doing the right things, whereas management is responsible for translating this vision into coordinated action by ensuring efficiency and effectiveness in execution, or doing things right.</td></tr>
<tr><td>Question 8
To what extent does the team responsible for leading the change demonstrate sufficient diversity of competencies across domains such as political acumen, strategic planning, people management, adaptive learning, philosophical reflection, and crisis management?</td><td>Options:
a. Political acumen: Yes or No
b. Planning skills: Yes or No
c. People management skills: Yes or No
d. Learning skills to support trial and error: Yes or No
e. Philosophical skills to be self-critical: Yes or No
f. Skills for crisis management: Yes or No</td></tr>
<tr><td colspan="2">Rationale: An effective team for a change project requires a complementary mix of competencies distributed across its members. Core competencies typically include political acumen, strategic planning and execution, and people management. In addition, the capacity for adaptive learning through trial and error, as well as reflective engagement with the broader purpose and implications of the project from a more philosophical perspective, provides important added value. Finally, the ability to adopt an appropriate management approach during periods of crisis is, in some cases, essential for sustaining the change process.</td></tr>
<tr><td>Question 9
Are stakeholders affected by the change adequately motivated to support its implementation?</td><td>Options:
a. Yes
b. Partially
c. No</td></tr>
<tr><td colspan="2">Rationale: Employee motivation is commonly viewed as a central component of readiness for change and has been consistently linked to successful outcomes in organizational change efforts. Within hospital settings, stakeholders can be categorized into various groups, such as clinical specialties (e.g., internal medicine, surgery, radiology), professional disciplines (e.g., physicians, nurses, managers), and roles such as administrative and support staff. Although these groups are often interdependent, change initiatives frequently overlook stakeholders whose influence is perceived as less significant.</td></tr>
</table>

**Question 10**<br>Do the stakeholders affcted by the change possess the required training and competencies?	**Options:**<br>a. Yes<br>b. Partially<br>c. No
**Rationale:** Employee change readiness constitutes a critical enabling condition for the successful implementation of organizational change. While employees' understanding of and motivation for the change are important components of readiness, the capacity to effectively perform the tasks required by the change is equally essential. Accordingly, sustained attention to training and professional development is necessary to support effective change execution.	
**Question 11**<br>Are material resources like time, money and equipment well arranged?	**Options:**<br>a. Yes<br>b. Partially<br>c. No
**Rationale:** Kotter's change framework underscores the importance of removing barriers to action, including ensuring that sufficient time, financial resources, and material support are available to facilitate successful change outcomes. From a diffusion-of-innovation perspective, the availability of adequate time, financial means, and material infrastructure supports trialability and reduces perceived complexity, both of which are critical determinants of successful innovation adoption	
**Question 12**<br>Which statement best describes professionals affected by the change?	**Options:**<br>a. Professionals were already involved in its design<br>b. Professionals are involved in evaluation and improvement<br>c. Professionals only execute the change
**Rationale:** Change management in clinical settings must explicitly recognize the professional context in which it is embedded. To foster support and enhance the likelihood of successful outcomes, it is essential to involve professionals at an early stage by providing them with meaningful opportunities to influence the design, implementation, and iterative adaptation of initiatives based on monitoring and evaluation findings.	
**Question 13**<br>Do we sufficiently care for all stakeholders affected by the change?	**Options:**<br>a. Yes<br>b. Partially<br>c. No
**Rationale:** Careful consideration of the side effects experienced by groups affected by change is essential for fostering support and enhancing the likelihood of successful change outcomes.	
**Question 14**<br>How detailed are the instructions for implementing the change?	**Options:**<br>a. Very loosely defined, since change is expected to happen mainly organically<br>b. A broad overview with room for professionals to fill in<br>c. Very detailed. All affected know exactly what to do
**Rationale:** Professionals generally value autonomy and trialability, particularly the opportunity to identify and implement solutions in ways they consider most appropriate for achieving agreed-upon objectives. Excessively detailed instructions, although sometimes requested, may have a demotivating effect and undermine professional engagement. Conversely, reliance on entirely organic change processes entails significant risk, as it raises the question of why such change would emerge at this moment if it has not occurred previously. Accordingly, we recommend a careful analysis of the organizational context to determine an appropriate level of instructional detail that balances guidance with professional autonomy.	
**Question 15**<br>How do affected professionals generally judge profit versus loss due to the change?	**Options:**<br>a. Profit > loss<br>b. Profit and loss in equilibrium<br>c. Loss > profit
Rationale: Kahneman showed that individuals tend to be highly sensitive to experiences of loss, whereby even relatively minor perceived losses can outweigh larger anticipated gains. Consequently, when the perceived losses associated with a change initiative exceed the perceived or expected benefits, the probability of successful implementation decreases. In particular, losses perceived by influential stakeholders like physicians may pose a significant challenge in a change project that requires careful people management.	
**Question 16**<br>To what extent does the organization foster an open culture of feedback and	**Options:**<br>a. Yes<br>b. Partially

improvement, and possess the capacity to address critical voices in a constructive manner?	c. No
**Rationale:** Agile change approaches emphasize the pursuit of a clearly articulated goal in combination with iterative cycles of feedback, critical reflection, and adaptation. Such continuous learning processes allow organizations to respond effectively to uncertainty and complexity, thereby enhancing the effectiveness of change initiatives.	
**Question 17**<br>During the change, speaking up is rewarded.	**Options:**<br>a. Yes<br>b. Partially<br>c. No
**Rationale:** Amy Edmondson conceptualized psychological safety as a shared belief that individuals can speak up, share ideas, and take interpersonal risks without fear of negative consequences. Empirical evidence from Google (Project Aristotle) demonstrated that psychological safety was a key enabling factor for team effectiveness and innovative performance. On this basis, we recommend that organizations actively encourage and reward employee voice, including the expression of ideas, perspectives, and strategic visions. The establishment of a psychologically safe environment constitutes a necessary precondition for fostering such a culture.	
**Question 18**<br>How is resistance to change addressed?	**Options:**<br>a. Through dialogue and room for individual choices<br>b. Through dialogue and persuasion<br>c. Through enforcement of the mandatory change
**Rationale:** Kotter's framework addresses resistance to change through the creation of urgency, the establishment of a guiding coalition, the communication of a clear vision, and the removal of structural and psychological barriers. Nevertheless, resistance may persist, necessitating the deliberate selection of an appropriate communication strategy. Dialogical approaches are generally preferable, particularly in professional contexts characterized by high levels of autonomy, such as hospital settings. Dialogue supports vision alignment and promotes behavioral change grounded in shared understanding and commitment. In contrast, enforcement may result in compliance without internalized acceptance, increasing the risk of superficial adherence and circumvention of the intended change. Consequently, enforcement is typically regarded as a strategy of last resort. While it may be justified in situations of high urgency, legal obligation, or persistent obstruction rooted in vested interests, its use entails risks to trust, commitment, and psychological safety and should therefore be applied selectively, proportionately, and in combination with clear communication and supportive measures.	
**Question 19**<br>What monitoring indicators are used?	**Options:**<br>a. Contented customers<br>b. Professionals executing the desired change-related routines.<br>c. Professionals able to execute the change.<br>d. Contented professionals
**Rationale:** Within the Plan-Do-Check-Act cycle, systematic monitoring constitutes a central mechanism for organizational learning and adaptive improvement. During the Check phase, performance is assessed against intended objectives; however, in practice, monitoring frequently relies on proxy indicators rather than direct measures of the ultimate change goals. Indicators that more closely approximate the intended outcomes enhance the validity of evaluation and support more informed decision-making in the Act phase. Nevertheless, when direct measurement is not feasible, the pragmatic use of well-chosen proxy measures represents an acceptable and often necessary approach to sustaining iterative learning and continuous improvement.	
**Question 20**<br>How is the change consolidated?	**Options:**<br>a. Trust in professional ownership<br>b. By inescapable routines on the work floor<br>c. By rewarding the planned behavior<br>d. By management driven monitoring and improvement cycles<br>e. By external monitors supported by legislation
**Rationale:** In healthcare settings, the sustainability of change depends on the extent to which new practices become normalized within everyday clinical work. Drawing on Normalization Process Theory, consolidation is understood as a social and collective process through which clinicians integrate new ways of working into existing professional routines. This process requires shared sense-making regarding the purpose and value of the change (*coherence*), sustained professional engagement and ownership (*cognitive participation*), alignment of roles, workflows, and resources (*collective action*), and ongoing appraisal of the change in practice (*reflexive monitoring*). In highly professionalized healthcare environments, normalization is facilitated by trust, professional autonomy, and the compatibility between the	

change and established clinical routines. Where these conditions are insufficiently addressed, change initiatives are likely to remain fragile and vulnerable to erosion or reversal over time.
